# Abiotic Stresses Elicitation Potentiates the Productiveness of Cardoon Calli as Bio-Factories for Specialized Metabolites Production

**DOI:** 10.3390/antiox11061041

**Published:** 2022-05-24

**Authors:** Rosa D’Alessandro, Teresa Docimo, Giulia Graziani, Vincenzo D’Amelia, Monica De Palma, Elisa Cappetta, Marina Tucci

**Affiliations:** 1 Institute of Bioscience and BioResources, National Research Council, Via Università 100, 80055 Portici, Italy; rosa.dalessandro@crea.gov.it (R.D.); vincenzo.damelia@ibbr.cnr.it (V.D.); monica.depalma@ibbr.cnr.it (M.D.P.); elisa.cappetta@ibbr.cnr.it (E.C.); mtucci@unina.it (M.T.); 2 Department of Pharmaceutical Science, University of Naples Federico II, Via Montesano, 80131 Napoli, Italy; giulia.graziani@unina.it

**Keywords:** cellular agriculture, abiotic elicitors, bioactive metabolites, plant cell extracts, cosmeceutics

## Abstract

Cultivated cardoon (*Cynara cardunculus* L. var *altilis*) is a Mediterranean traditional food crop. It is adapted to xerothermic conditions and also grows in marginal lands, producing a large biomass rich in phenolic bioactive metabolites and has therefore received attention for pharmaceutical, cosmetic and innovative materials applications. Cardoon cell cultures can be used for the biotechnological production of valuable molecules in accordance with the principles of cellular agriculture. In the current study, we developed an elicitation strategy on leaf-derived cardoon calli for boosting the production of bioactive extracts for cosmetics. We tested elicitation conditions that trigger hyper-accumulation of bioactive phenolic metabolites without compromising calli growth through the application of chilling and salt stresses. We monitored changes in growth, polyphenol accumulation, and antioxidant capability, along with transcriptional variations of key chlorogenic acid and flavonoids biosynthetic genes. At moderate stress intensity and duration (14 days at 50–100 mM NaCl) salt exerted the best eliciting effect by stimulating total phenols and antioxidant power without impairing growth. Hydroalcoholic extracts from elicited cardoon calli with optimal growth and bioactive metabolite accumulation were demonstrated to lack cytotoxicity by MTT assay and were able to stimulate pro-collagen and aquaporin production in dermal cells. In conclusion, we propose a “natural” elicitation system that can be easily and safely employed to boost bioactive metabolite accumulation in cardoon cell cultures and also in pilot-scale cell culture production.

## 1. Introduction

Plants produce bioactive molecules for human health through their specialized metabolism. Extracts containing these compounds are increasingly utilized in nutraceutical, pharmaceutical, and cosmetic formulations. Production of phyto-ingredients from cultivated plants can be environmentally and ethically costly due to unsustainable agricultural practices that make use of large amounts of fertilizers, pesticides, water and energy to maximize yield [1], and to subtraction of cultivated land from food and feed making. Moreover, industrial production requires quantitatively and qualitatively constant availability of raw materials, which contrasts with the seasonality of plant cultivation.

Plant cell cultures could be an effective alternative for producing a potentially unlimited supply of bioactive extracts with standardized composition in an eco-friendly manner, devoid of the typical limits of seasonal variability that characterize field-cultivated plants. The versatility of this system also allows the use of elicitation to increase the yield of certain specialized metabolites [2,3,4]. An elicitor can be a chemical or physical factor that mimics biotic or abiotic stress by activating signal transduction cascades, including expression of genes related to the biosynthesis of specialized metabolites, which ultimately determines the increase in defensive bioactive compounds. Biotic-based elicitation usually employs molecules, microbial-derived or defensive messenger molecules, that are recognized by plant cells as a pathogen attack [3]. Likewise, the use of abiotic elicitors, such as salinity, temperature, drought, and UV light, can stimulate the plant’s enzymatic machinery leading to the production of molecules with antioxidants or osmoprotectant properties [5,6]. The efficacy of elicitation for a specific target compound depends on the plant species, genotype, and tissue, and not least by the growth conditions (e.g., temperature, medium composition, and light) [7]. As an example, elicitation of *Catharanthus roseus* cell cultures with extracts of endophytic fungi allowed production of vinblastine and vincristine, an important class of tropane indol alkaloids, which was not previously been achieved in cultured cells [8]. Similarly, undifferentiated callus tissues of *Cannabis sativa*, even those derived from flowers, were not able to synthesize cannabinoids, unless methyl jasmonate was added to the medium in combination with the precursor [9,10]. Moreover, several cell cultures are able to produce, along with desired metabolites, other novel compounds, mostly due to the presence of active metabolites in various isomeric forms, that are unusual in the original plant [1]. These metabolic changes are often consequence of elicitation, as reported for hispidol in *Medicago truncatula* [11,12]. Elicitation of cell cultures can effectively provide substrate pools and catalysts for the generation of novel metabolites but often limits biomass formation as observed for example for Taxus cells [13,14]. All the above aspects should be taken into account before planning the development of cell cultures for producing desired compounds or setting up elicitation strategies.

Our group has long been interested in the nutraceutical and health-promoting properties of *Cynara cardunculus*, among which cultivated cardoon (*Cynara cardunculus* L. var. *altilis*) [15,16,17,18], an orphan crop exploited in several Mediterranean regions as traditional food and a vegetable coagulant for cheese production [19,20]. More recently, we have also addressed cardoon as an oleaginous and lignocellulosic biomass species [15,16], whose cultivation has been valorized as a sustainable, renewable source of raw materials for bioplastics and biofuels production [21,22,23,24]. Several cardoon varieties are able to maintain growth and yield even under unfavorable environmental conditions and we observed that leaves of the genotype ‘Spagnolo’ accumulate high amounts of different bioactive phenylpropanoids [15], especially during salinity stress [16]. Basing on these previous lines of evidence, we hypothesized that abiotic elicitors could drive a successful production of antioxidant metabolites in cardoon derived calli culture with a limited negative impact on the cellular growth. In order to ascertain whether metabolic changes at the cellular level could resemble the whole plant response, we applied chilling and salt stress as eustress on cardoon calli. Moreover, the present research was intended to explore the use of ‘Spagnolo’ cells as bio-factories for bioactive phenylpropanoids. The elicitation (stress) experiments also gave us the opportunity to produce data, though to a limited extent, related to the cardoon cellular response to salt and chilling stress and to evaluate the effectiveness of the use of these abiotic “natural” elicitors for boosting bioactive molecule production. Finally, elicitated calli extracts were selected for evaluation of their cosmeceutical potential.

## 2. Materials and Methods

### 2.1. Plant Material and Callus Induction

Callus cultures were established according to [25] and maintained in continuous dark conditions at 24 °C in a growth chamber. Once established, callus cultures were subcultured every 30 days on agarized Gamborg B5 medium with the same composition as for induction. After several subcultures on the optimized callus medium, vigorously growing callus cultures (Supplementary Figure S1A,B) were selected for growth characteristic assessment and further analysis.

### 2.2. Experimental Design and Stress Treatments

#### Chilling and Salt Stress

In order to evaluate the effects of chilling stress on growth rate and metabolism of cardoon calli, a weighed amount of cardoon callus was placed in GB5 medium and maintained in a growth chamber in dark conditions at 6 °C for 7 and 14 days for chilling stress, while control calli were maintained at 24 °C.

For salt stress treatment, cardoon calli were weighed at time 0 and placed in GB5 medium supplemented with 0 mM, 50 mM, 100 mM, or 150 mM NaCl for 14 and 28 days in a growth chamber at 24 °C. After 14 and 28 days, calli were weighed for growth evaluation.

For biochemical and molecular analyses, stressed and unstressed calli at each time point were frozen under N_2_, and stored at −80 °C.

### 2.3. Growth Evaluation

Callus growth was evaluated by measuring fresh weight (g) of the callus in control and stressed conditions at each time point of the treatment. A total of three biological replicates (each consisting of three calli *n* = 9) were used for each treatment. The weight was recorded at T0, corresponding to initial transfer on fresh medium, as well as after 7 and 14 days for chilling stress, and 14, and 28 days for salt stress. Growth rate was calculated as mean callus relative growth rate (GR) by [(T final − T initial)/T initial] × 100% according to what reported by [26].

### 2.4. Ultrasound Assisted Extraction of Polyphenols and UHPLC-Q-Orbitrap HRMS Analysis

Polyphenols were extracted from 3 g of lyophilized calli following the methods detailed in [15]. The ultrasonic calli extraction procedure was repeated three times. Polyphenols were quantified and separated using an UHPLC system equipped with a quaternary pump (Ultimate 3000, Thermo Fisher Scientific, Waltham, MA, USA), and a Kinetex 2.6 µm Biphenyl (100 × 2.1 mm, Phenomenex, Torrance, CA, USA) column. Mass spectrometry analysis was carried out with a Q Exactive Orbitrap LC-MS/MS (Thermo Fisher Scientific, Waltham, MA, USA).

The acquisition of polyphenolic compounds was performed according to [15], where the protocol is fully detailed. The calibration and accuracy of the equipment were monitored by using a reference standard mixture (Thermo Fisher Scientific, Waltham, MA, USA). Data acquisition and processing were performed with Quan/Qual Browser Xcalibur software, v. 3.1.66.10 (Xcalibur, Thermo Fisher Scientific, Waltham, MA, USA).

### 2.5. RNA Extraction and Expression Analysis

Total RNA was extracted from 100 mg of liquid nitrogen-ground control and stressed calli using the RNeasy kit (Qiagen, Chatsworth, CA, USA), and quantified using a NanoDrop ND-8000 spectrophotometer (Thermo Fisher Scientific, Wilmington, DE, USA). A sample of one μg of total RNA was reversed transcribed using the QuantiTec Reverse Transcription Kit (Qiagen, Chatsworth, CA, USA), according to the manufacturer’s instructions. Real-time qRT-PCR was performed with Power up^®^ SYBR^®^ Green qPCR Super Mix (Applied Biosystem, Foster City, CA, USA) in a ABI7900 HT (Life Technologies, Foster City, CA, USA). Each PCR reaction (20 μL) contained 10 μL real-time qRT-PCR Mix, 4 μL of a 1:25 dilution of cDNA, and 0.25 μM of each specific primer designed from sequences retrieved from the *C. cardunculus* genome (Annotation v2.0 assembly [27]). The thermal cycling conditions were 50 °C for 2 min, 95 °C for 2 min, followed by 40 cycles of 15 s at 95 °C and 30 s at 60 °C. PCR product melting curves were analyzed for the presence of a single peak. All reactions were performed on biological triplicates and technical duplicates. Fold change measurements were calculated with the 2^−ΔΔCT^ method [28,29]. For each time and treatment, expression of calli in control conditions was used as internal calibrator and then set equal to 1. Gene expression was normalized to the stably expressed cardoon actin gene.

Sequences of primers used for real-time qRT-PCR for *CcHQT* (Hydroxycinnamoyl quinate transferase (DQ915589.1)) and *Ccactin* (XM_025103545) were previously reported [15] and for *CcF3′H* (Forward primer 5′-CCTGCAAAGCGTGACGAAGA-3′ and 5′-ACGACGTCACCGCCATTTTT-3′ Reverse primer) designed on HM153534 as reported [18].

### 2.6. Antioxidant Activity: ABTS, DPPH, and FRAP Assays

Determination of the ABTS (2,2′-azino-bis (3-ethylbenzothiazoline-6-sulfonic acid)) free radical scavenging activity was carried out following the method described by [30]. Briefly, 44 µL of aqueous potassium persulfate (2.45 mM) were added to 2.5 mL of aqueous ABTS (7 mM) and incubated in the dark at room temperature (23 °C) for 12–16 h. This ABTS stock solution was diluted (1:88) with ethanol to obtain a working solution with an absorbance of 0.700 ± 0.050 at 734 nm. The assay was performed by adding 0.1 mL of filtered and properly diluted sample to 1 mL of ABTS^+^ working solution and the absorbance was read at 734 nm after 2.5 min. Results were expressed as Trolox^®^ equivalent antioxidant capacity (TEAC, mmol Trolox^®^ equivalents Kg-1 dry weight of plant). All determinations were performed in triplicate.

The DPPH (2,2-diphenyl-1-picrylhydrazyl) free radical-scavenging activity was determined using the method proposed by [31] with few modifications. Briefly, 100 μM (1 mL) of DPPH radical methanolic solution was added to polyphenol extract (0.2 mL) and the absorbance of the resulting solution was spectrophotometrically read at 517 nm after 10 min. The activity was expressed as Trolox^®^ equivalent antioxidant capacity (TEAC, mmol Trolox^®^ equivalents Kg^−1^ dry weight of plant). All determinations were performed in triplicate.

The ferric reducing antioxidant activity was evaluated using a FRAP (ferric ion reducing antioxidant power) assay [32] with a few modifications. The FRAP reagent was made up of 10 μM TPTZ (2,4,6-tris (2-pyridyl)-1,3,5-triazine) in 40 μM HCl, 20 μM of aqueous FeCl_3_ and acetate buffer (300 μM, pH 3.6) at 1:1:10 (*v*/*v*/*v*). Sample solutions, properly diluted (10 μL), and FRAP reagent were mixed and the absorbance was monitored at 593 nm after 10 min. The results were expressed in mmol Trolox^®^ Kg^−1^ dry weight (DW). The results were corrected for dilution and expressed as Trolox^®^ equivalent antioxidant capacity (TEAC, mmol Trolox^®^ equivalents Kg^−1^ dry weight of plant). All determinations were performed in triplicate.

### 2.7. Lipid Peroxidation Measurement

Lipid peroxidation was estimated by determining the malonyldialdehyde (MDA) contents in calli as previously reported [16]. One hundred milligrams of samples were homogenized in 2 mL of 0.1% trichloroacetic acid (TCA). The homogenate was centrifuged at 15,000× *g* for 10 min at 4 °C. A 0.5 mL aliquot of the supernatant was mixed with 1.5 mL of 0.5% thiobarbituric acid (TBA) prepared in TCA 20%, and incubated at 90 °C for 20 min. After stopping the reaction in an ice bath, samples were centrifuged at 10,000× *g* for 5 min. The supernatant absorbance at 532 nm was then measured. After subtracting the non-specific absorbance at 600 nm, MDA concentration (three replicates per treatment) was determined using the extinction coefficient 155 mM^−1^ cm^−1^.

### 2.8. Human Cell Cultures

Human Dermal Fibroblasts (HDF), purchased by (ThermoFisher Scientific Catalog C-004-SC) derived from neonatal foreskin and immortalized human keratinocytes HaCaT (AddexBio Technologies, Catalog No. T0020001) were maintained in Dulbecco’s Modified Eagle Medium (DMEM) supplemented with 10% of fetal bovine serum in 95% air, 5% CO_2_ and humidified atmosphere at 37 °C.

### 2.9. MTT Assay

A total of 9 × 103 HaCaT were seeded in 96-well plates, grown for 8 h, and treated for 48 h with different concentrations of cardoon cell extracts. After treatments, cells were washed with PBS and incubated with 100 μL per well of reaction buffer containing the following: 10 mM Hepes, 1.3 mM CaCl_2_, 1 mM MgSO_4_, 5 mM glucose, and 0.5 mg mL^−1^ of colorimetric substrate MTT [3-(4,5-dimethylthiazolyl)-2,5-diphenyltetrazolium bromide] in PBS buffer at pH 7.4, according to the method described by [33]. After 3 h at 37 °C, cells were solubilized by the addition of 100 μL solubilization solution (TritonX100, 0.1N HCl in 10% isopropanol), and the plate was incubated for 4 h at room temperature. The number of healthy cells is directly proportional to the level of the formazan product created. The developed color was then quantified at 595 nm using a multiwell plate reader (Victor Nivo Perkin Elmer).

### 2.10. Hydrating Power

Hydrating power by Elisa determination of Aquaporin-3 (AQP3) content was tested on HaCaT cells. HaCaT cells were seeded at a density of 10 × 10^3^ in 96-well plates and grown for 16 h, then incubated with cardoon calli extracts for 24 h at 37 °C. After the treatments, the medium was removed and the cells were washed with PBS 1x, fixed in paraformaldehyde (PFA) 4% for 10 min and then incubated for 1 h with BSA 3% to saturate non-specific bonds. The cells were then incubated for 2 h with primary antibody, raised against AQP3 (PA5-53257 Thermo Fisher) dissolved in PBS with BSA 1%. After 3 washes, the secondary antibody (antirabbit 170-6515 Biorad) diluted in PBS with BSA 1% was added and incubated for 1 further hour. Plates were washed 3 times, one hour later, with PBS 1x and the amount of AQP3 produced by the cells was measured by a colorimetric reaction, using 0.35 mg/mL of o-phenylenediamine (OPD) (Sigma-Aldrich, St. Louis, MO, USA) and 0.012% of H_2_O_2_ in citrate buffer 50 mM. The developed color was measured by reading the absorbance at 490 nm using the multiplate reader Victor Nivo (Perkin Elmer, Waltham, MA, USA). The signal for each sample was compared to cellular density determined by crystal violet staining (Sigma-Aldrich, St. Louis, MO, USA).

### 2.11. Pro Collagene I Production

HDF cells were seeded at a density of 8 × 103 in 96-well plates and treated with cardoon calli extracts or transforming growth factor-β (TGFβ) (2.5 ng/mL) as positive control. After the treatments, the medium was removed and the cells were washed with PBS 1x, fixed in paraformaldehyde (PFA) 4% for 10 min, and then incubated for 1 h with BSA 3% to saturate non-specific bonds. The cells were then incubated for 2 h with ProCollagen I specific antibody (sc-166572 Santa Cruz Biotechnology, Dallas, TX, USA) diluted 1:500 in PBS 1x, containing 1% BSA. After 3 washes, secondary antibody (anti-mouse 170-6516 Biorad) diluted in PBS containing 1% of BSA was added. Plates were washed 3 times with PBS 1x, one hour later, and the amount of ProColI produced by the cells was measured by a colorimetric reaction, using a 0.35 mg mL^−1^ solution of o-phenylenediamine (OPD) (Sigma-Aldrich, St. Louis, MO, USA) and 0.012%  of H_2_O_2_ in citrate buffer 50 mM. The developed color was measured by reading the absorbance at 490 nm using the multiplate reader Victor Nivo (Perkin Elmer, Waltham, MA, USA). The signal for each sample was compared to cellular density determined by crystal violet staining (Sigma-Aldrich, St. Louis, MO, USA).

### 2.12. Statistical Analysis

Results of growth are presented as mean ± standard deviation (SD) (*n* = 9). Quantitative results for metabolic analyses, qRT-PCR analyses, MTT, and Elisa assays were expressed as the mean value ± SD (*n* = 3). Significant differences for all the data were determined by comparing mean values through a factorial analysis of variance (ANOVA) with Tukey’s post hoc test at a significance level of 0.05. A non-clustered heatmap was generated by using the http://biit.cs.ut.ee/clustvis (accessed on 20 March 2022) program package.

## 3. Results

### 3.1. Calli Growth under Stress Conditions

**Figure 1:**
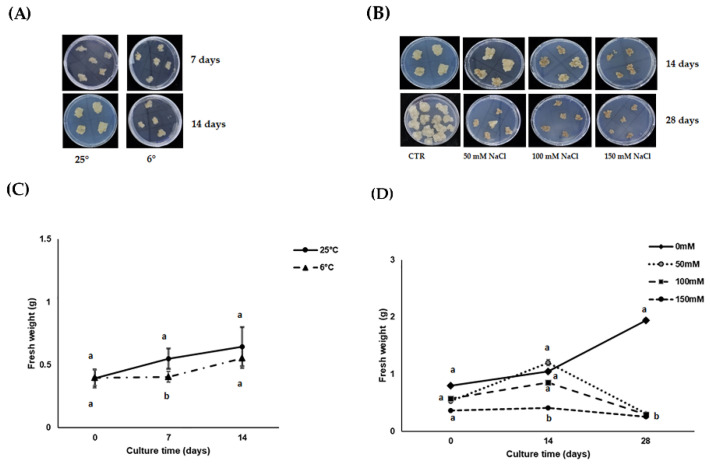
(**A**–**D**) Appearance of cardoon calli on solid Gamborg B5 culture medium under (**A**) chilling stress at 6 °C for 7 and 14 days or (**B**) saline stress of 0, 50, 100 or 150 mM NaCl for 14 and 28 days. Growth changes of cardoon calli subcultured on solid Gamborg B5 culture medium under (**C**) chilling stress at 6 °C for 7 and 14 days or (**D**) saline stress of 0, 50, 100 or 150 mM NaCl for 14 and 28 days. Each value represents the mean of nine biological replicates ± SD/10. Statistical significance between samples within each time point was determined by one-way ANOVA with Tukey’s post hoc test. Different letters indicate significant differences (*p* < 0.05).

To test whether the chosen abiotic stress might compromise calli growth, we compared the fresh weights of calli subjected to either chilling or salt stress with that of respective controls (growth at normal conditions i.e., at 25 °C and/or without salt). No evident signs of stress were observed in cardoon calli under chilling stress, at least on visual appearance (Figure 1A). On the contrary, calli cultivated under salt stress showed toxicity symptoms (i.e., browning and growth reduction) either under high salt imposition or prolonged stress (Figure 1B). As regards growth under chilling stress, we measured a growth rate reduction of about 38% after 7 days of chilling imposition. Unstressed calli showed a growth increase of 39.8%, whereas stressed calli were about 1.8%. By extending the chilling treatment to 14 days, we observed that calli completely recovered their regular growth, as the weight was comparable to control unstressed calli at 25 °C. Namely, unstressed calli had a growth rate of about 64%, whereas chilled calli showed a growth rate increase of 40%, but were not statistically different from one another (Figure 1C). As regards salt stress, we used three different concentrations of NaCl. After 14 days, calli at the highest NaCl concentration (150 mM) increased their growth rate by only 13% as compared with the respective initial weight (~0.36 g vs. ~0.41 g), whereas at 50 and 100 mM NaCl, the fresh weight almost doubled compared to the initial weight (~0.52 g and ~0.57 g vs. 1.1 g and 0.98 g, respectively) with a growth rate of 127% and almost 50%, but this increase was not statistically different from calli growing in control conditions (Figure 1D). However, a longer exposure (28 days) to any salt concentration completely impaired calli growth. While at 28 days the fresh weight of control calli increased about 2.5 times higher than the initial weight (~0.8 g at T0 vs. 1.94 g at 28 days), NaCl-stressed calli showed half reduced growth compared to controls (Figure 1D). Overall, the severity of growth reduction seemed to be more related to the stress duration than to the concentration of salt.

### 3.2. Chilling and Salt Stresses Elicit Phenylpropanoid Accumulation

#### 3.2.1. Metabolic Changes

Since environmental stresses are known to redirect plant metabolism towards the production of molecules having defense and signaling functions, chilling and salinity might be good elicitors for the biosynthesis of specialized metabolites. Therefore, we monitored changes in phenylpropanoid compounds, such as mono- or di-caffeoylquinic acid derivatives commonly found in plants of the Asteraceae. Based on the LC-MS analysis, 17 different phenolic acids, mainly caffeoyl quinic derivatives and flavonoids, were identified in cardoon callus cultures in response to 7 and 14 days of chilling stress at 6 °C (Table 1). Differences between control and stressed calli were merely quantitative because a similar phenolic profile was observed and no peaks from new compounds were observed. As shown in Table 1, chilling affected the production of almost all the detected hydroxycinnamic acids (e.g., 3-CQA, 3-caffeoyl quinic acid [chlorogenic acid, CGA]; 3-FQA, 3-feruloyl quinic acid; 5-FQA, 5-feruloyl quinic acid; 3,4-DiCQA, 3,4-dicaffeoyl quinic acid; 1,5-DiCQA, 1,5-dicaffeoylquinic acid [cynarin]; 5-iFQA 5-O-isoferuloylquinic acid; p-coumaric acid) while no statistical effects were observed for flavonoids with the exception of quercetin-glucosides. The eliciting power of 7-day chilling compared to the control resulted in: (1) an almost 20-fold increase in the concentration of chlorogenic acid; (2) about one-fold increase in CGA-related dimeric forms (3,4-DiCQA, 1,5-DiCQA); (3) 4-fold increase in p-coumaric as well as the only flavonoid quercetin glucosides (Table 1). Prolonged chilling stress of 14 days did not have a proportionally higher stimulation of phenolic compounds, which resulted in similarly triggered as at 7 days or even slightly less (e.g., 5-FQA, 1,5-DiCQA and 5-iFQA) (Table 1). Chilling stress stimulated production of total phenols and this increase was mostly related to a higher amount of chlorogenic and di-chlorogenic acids and their precursor p-coumaric acid, which significantly increased compared to 14-day unstressed calli, but not as much as in 7-day stressed calli (Table 1).

Saline stress strongly triggered phenylpropanoid accumulation (Table 2). The level of total phenols at any dose and timing of salt imposition was significantly higher than in control conditions. After 14 days at 100 mM, total phenols of NaCl-stressed calli reached 8301.521 ± 234.433 ppm, higher than the highest level of total phenol obtained with chilling stress (i.e., 7289.527 ± 211.430 after 7 days at 6 °C). Notably, both at 14 and 28 days, the amount in CGA, CGA dimers, and quinic acids increased proportionally to the NaCl dose. In particular, after 14 days of salt stress, CGA amount increased three- to six-fold with NaCl concentration compared to unstressed calli. A similar trend was found for dicaffeoyl quinic acids (3,4-DiCQA and 1,5-DiCQA) and for the feruloyl quinic acids (3-FQA and 5-FQA), for which an average increase of 1.8 times compared to the control was observed. Finally, an increase of about three times was observed with saline treatment (150 mM) for the flavonoid quercetin glucoside while no significant changes were observed in minority compounds.

Similarly, after 28 days of salt stress, the treatment with a salt concentration of 150 mM caused an increase in polyphenol biosynthesis. In particular, an average increase of about 2.5 times was observed for the most represented compounds (diCQAs, FQAs, and quercetin glucoside), excluding chlorogenic acid, which showed an increase of about 30 times compared to the control.

#### 3.2.2. Transcriptional Changes

**Figure 2:**
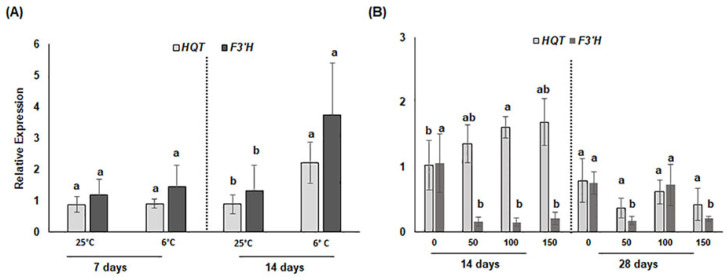
(**A**,**B**) Relative expression changes of *CcHQT* and *CcF3′H* biosynthetic genes in cardoon callus cultures in response to (**A**) chilling stress for 7 and 14 days at 6 °C or (**B**) saline stress at 0, 50, 100 or 150 mM NaCl for 14 and 28 days. Total RNA was extracted from unstressed (CTR) and treated calli (chilling or salt stress) and unstressed calli at each time point were used as controls. Each value represents the mean of three biological replicates ± SD. Statistical significance for each gene between treatments within each time point was determined by using one-way ANOVA with Tukey’s post hoc test. Different letters indicate significant difference (*p*-value < 0.05).

To understand whether transcriptional activity was correlated with phenylpropanoid production and accumulation upon abiotic stress imposition, we evaluated the expression levels of two key structural genes involved in the biosynthesis of hydroxycinnamic acids and flavonoids, namely *HQT* (Hydroxycinnamoyl quinate transferase) and *F3′H* (Flavonoid 3′hydroxylase), involved in chlorogenic acid formation and hydroxylation of the 3′ position of the flavonoids B-ring, respectively. After 7 days of chilling stress, no significant transcriptional changes were detected either for *CcHQT* or *CcF3′H* between stressed and unstressed calli. Instead, after 14 days, a significant increase of about two-fold for the transcripts of both genes was detected in chilled compared to unstressed calli (Figure 2A,B).

Regarding saline treatment, after 14 days of the treatment *CcHQT* transcripts were significantly up regulated with respect to the control only at 100 mM NaCl, whereas *CcF3′H* transcript levels were significantly reduced at all NaCl concentrations. After 28 days of salt stress, the expression levels of both genes were comparable or significantly lower (at 50 and 150 mM) than in control calli. Our expression analysis shows that transcription of both genes is influenced by these abiotic stresses, though a weak correlation can be detected with biochemical data (Table 1 and Table 2), and this highlights that expression changes can be only indicative of the transcriptional dynamics linked to CQA and flavonoid accumulation but not of their effective metabolic flux, which necessarily requires the integration of different kinds of omics data.

### 3.3. Changes in Antioxidant Activities

**Figure 3:**
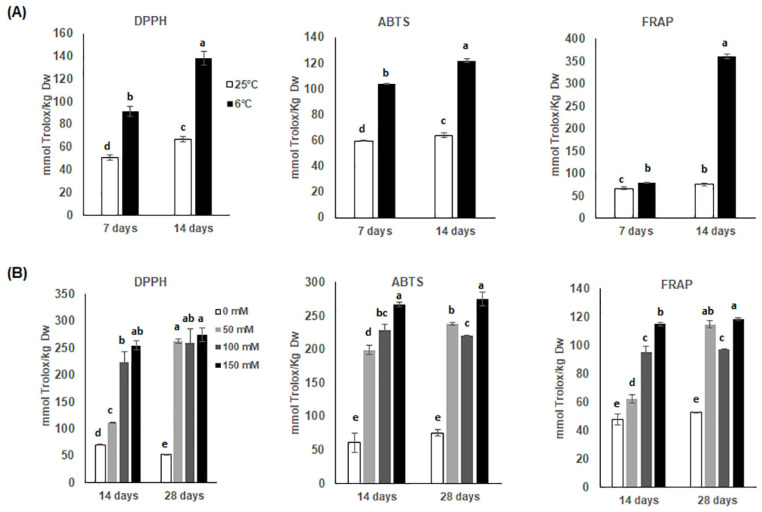
(**A**,**B**) Changes in antioxidant power measured via 2,2-diphenyl-1-picrylhydrazyl (DPPH), 2,2’-azino-bis 3-ethylbenzothiazoline-6-sulfonic acid (ABTS), and ferric reducing antioxidant power (FRAP) assays in cardoon callus cultures in response to (**A**) 7–14 days chilling at 6 °C or (**B**) 14–28 days of 0, 50, 100, or 150 mM NaCl stress. Values are expressed as TEAC (Trolox^®^-equivalent antioxidant capacity, mmol Trolox Kg^−1^ DW). Each value represents the mean of three biological and two technical replicates ± SD. Statistical significance for each activity between treatments within each time point was determined by using one-way ANOVA with Tukey’s post hoc test. Different letters indicate significant difference (*p*-value < 0.05).

The results obtained for antioxidant activity of cardoon calli samples through the ABTS, FRAP and DPPH assays are presented in Figure 3 and expressed as TEAC (mmol Trolox^®^ equivalents Kg^−1^ DW). All extracts of stressed calli showed a higher antioxidant power than control calli. The levels of antioxidant activity of chilled calli extracts obtained via DPPH and ABTS assays both at 7 and 14 days were comparable, with 70–125% increments over the corresponding unstressed controls, whereas the FRAP assay indicated a 7-fold increment of the antioxidant power of the chilling-stressed calli extracts only after 14 days (Figure 3A). Regarding salt stress, both 14 and 28 days of stress showed a similar antioxidant eliciting power (Figure 3B). In these conditions, DPPH and ABTS showed similar results for the three NaCl concentrations, whereas for both time points the FRAP assay revealed an increase in TEAC that was half of that measured with ABTS and DPPH for the different NaCl levels.

Since chlorogenic acids and di-caffeoylquinic acids are the main polyphenolic components in the extracts, these results suggest that they are mostly responsible for the radical scavenging activities of cardoon. This is supported by the observation that the content increase in these phenolics strongly correlates with increase in DPPH and ABTS values. Namely, under chilling stress a perfect positive correlation is detected for DPPH and ABTS activities. Similarly, under 14- and 28-day salt stress, highly positive correlations between total phenols and DPPH as well as ABTS (R = 0.893 and R = 0.990; R = 0.982 and R = 0.942), respectively, were found at the different NaCl concentrations. Regarding the FRAP assay, which measures the reduction of the ferric ion (Fe^3+^) to ferrous ion (Fe^2+^) by donor electrons in the sample [34], cardoon extracts showed a very high FRAP activity after 14 days of chilling compared to all the other extracts. This effect cannot be associated with the polyphenol content in the extract but possibly to other molecules not detected in our analysis.

### 3.4. Effects of Stresses on Lipid Peroxidation of Cardoon Calli

To evaluate whether chilling or salinity could induce oxidative stress in cardoon calli, we measured lipid peroxidation by the MDA assay. The 6 °C treatment reduced MDA content at both time points, and significantly after 7 days, suggesting that chilling alleviates oxidative stress (Table 3). On the contrary, NaCl treatments raised lipid peroxidation, with a significant accumulation of MDA after 14 days, at 100 and 150 mM NaCl. Consistently, MDA levels increased with the NaCl dose after prolonged stress (28 days) (Table 3).

### 3.5. Evaluation of the Optimal Elicitation Strategy

**Figure 4:**
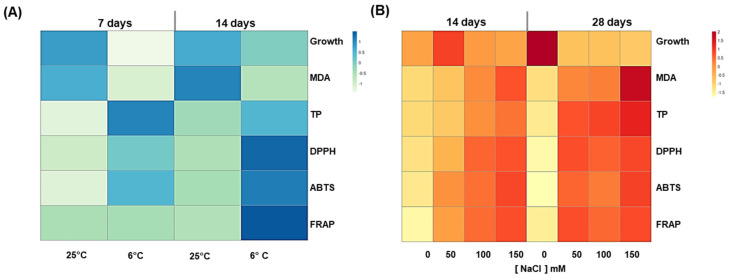
(**A**,**B**) Non-clustered heat map of major changes exerted by elicitation by abiotic stresses in cardoon calli. Heat maps for (**A**) 6 °C chilling stress treatment for 7 or 14 days, and (**B**) 0, 50, 100, or 150 mM NaCl salt stress treatment for 14 or 28 days. The columns of the heat maps represent control and stress treatments at the different time points, the rows represent different physiological and metabolic parameters (growth, lipid peroxidation MDA, total polyphenols, DPPH, ABTS, and FRAP assay). Each cell is colored based on the value in that sample. The intensity of color is representative of degree of differences; the value of 0 denotes no difference, with higher values corresponding to larger differences. Values from lower to higher are indicated by light green to blue for chilling stress and yellow to red for salt stress.

All the above analyzed parameters were useful to decide which stress conditions could exert the best elicitation of phenylpropanoids without compromising growth. In Figure 4A,B, the heat maps display the parameters that better represent the morphological, biochemical, and physiological changes detected in cardoon calli in response to the different stress conditions and timing. Looking at total phenolics (TP), the eliciting power was generally higher for salt than chilling stress. The antioxidant levels, measured by both ABTS and DPPH methods, mirrored the TP increase, although the rise of these antioxidant molecules was not enough to compensate for lipid peroxidation after long exposure (28 days) to salt stress, when a strong limitation of calli growth was observed. The best eliciting treatment was 100 mM NaCl for 14 days, which showed the highest accumulation of bioactive phenylpropanoids, along with a comparable growth to unstressed calli. Therefore, cardoon call treated with 100 mM NaCl for 14 days were selected for preparing hydroalcoholic extracts for the evaluation of biological activities, as reported below.

### 3.6. Cytotoxicity of Cardoon Calli Extracts

**Figure 5:**
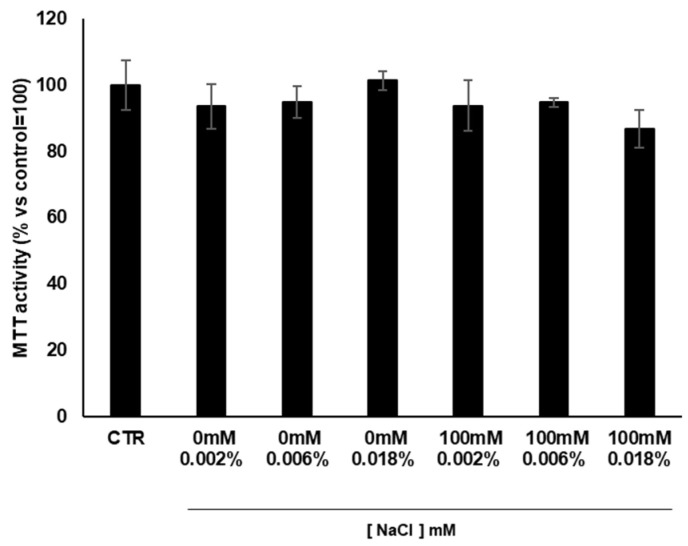
Protection activity of cardoon calli extracts on cell viability. Human keratinocytes were treated with the test compounds at different concentrations. The cells were processed as described in the Material and Methods section to measure cell viability (MTT assay). Each value represents the mean of three biological replicates ± SD. CTR, control unstressed cells; 0 mM and 100 mM NaCl, saline stress treatments.

Based on the above results, the selected 14-day 100 mM NaCl salt-stressed calli and respective control in unstressed conditions were analyzed for their biological activities. To test whether cardoon calli extracts could influence cell viability and to evaluate the extract concentrations to be used in the cellular tests, we measured the cytotoxic effects of the stressed and unstressed calli extracts on keratinocyte cell lines (HaCaT) via the MTT (3-(4,5-dimethylthiazol-2-yl)-2,5-diphenyltetrazolium bromide) cytotoxicity assay (Figure 5). HaCaT cells were treated with three increasing concentrations of the stressed and unstressed cardoon calli extracts (0.002, 0.006, and 0.018% *w*/*v*), and no cytotoxicity was detected at any of the used concentrations. For convenience, the 0.002% and 0.006% doses of the extract were chosen for the following experiments.

### 3.7. Analysis of Procollagen I and AQP3 Production

**Figure 6:**
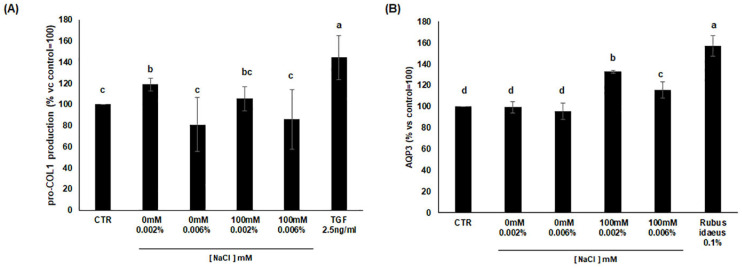
Effects of cardoon calli extracts on increasing the production of (**A**) pro-collagen I compared to TGFβ treatment and (**B**) AQP3 protein in comparison with *Rubus idaeus* as positive control; the quantization was performed by detection with antibody labeled with fluorophore. The bars represent the mean of three biological replicates ± SD. Statistical significance was determined between treatments within each time point by using one-way ANOVA with Tukey’s post hoc test. Different letters indicate significant difference (*p*-value < 0.05). CTR, control unstressed cells; 0 mM and 100 mM NaCl, saline stress treatment.

TGF-βs (Transforming Growth Factor-β) are shown to participate in wound healing and tissue repair and regulate the rate and duration of these processes. Following the TGFβ signal transduction activation, we analyzed whether cells treated with cardoon extracts might support the synthesis of procollagen type I in human dermal fibroblasts (HDF), which is one of the main proteinaceous components of the extracellular matrix. As shown in Figure 6A, the cardoon extract from unstressed calli increased the production of pro-collagen by about 20% only at the lowest concentration of 0.002%, while the same extract at higher concentration did not significantly affect pro-collagen production. Regarding extracts from 100 mM NaCl stressed calli, their effect was not significantly different from control at any concentration (Figure 6A).

To investigate the potential effect of the extracts on hydration, we evaluated the production of Aquaporin-3 (AQP3), a channel protein involved in the glycerol/water transport through membranes and in the maintenance of the water-proof capacity of the epidermis proteins specifically involved in the maintenance of water balance and collagen stability in human keratinocytes [35]. As a positive control of the assay, extract of *Rubus ideaus*, known for its hydrating power [35], was used comparatively. The results showed that extracts from 100 mM NaCl stressed cardoon calli increased the production of the AQP3 protein in comparison to the control (taken as 100%) (Figure 6B). More specifically, the extracts used at 0.002% and 0.006% were able to induce AQP3 by 30% and 15%, respectively. Our results proved the capacity of the cardoon extracts to act on AQP3 production and as modulating agents of skin hydration.

## 4. Discussion

Cellular agriculture, that is the production of agricultural commodities from cell cultures instead of field-cultivated whole plants, is an emerging biotechnology for the production of valuable molecules with a wide range of uses in the pharmaceutics, cosmetics, innovative materials, food and food additives, etc. industries [36,37,38,39,40,41,42]. Provided that plant cell bioreactors are designed to reduce inputs, especially energy, water, and waste, they can be an efficient and sustainable technique to save land and reduce agricultural footprints [25,42,43]. Moreover, cell cultures do not suffer from seasonal variation in yield and quality, typical of field production. A further advantage of plant cell cultures is that they can be stimulated in different ways for increasing the biosynthesis of bioactive metabolites, thus avoiding classical metabolic engineering or even the innovative CRISPR-Cas mediated strategies to manipulate the plant metabolism toward the production of valuable molecules [44], which are still not universally accepted by consumers and/or producers that want to invest in GMO-free cell platforms.

In this work, we used calli from leaves of *C. cardunculus* ‘Spagnolo’ with the aim of evaluating the efficacy of two strategies of abiotic elicitation in stimulating the accumulation of bioactive metabolites, while having a minimal perturbation effect on the growth of cardoon cell cultures. Albeit cardoon is a tolerant species able to also grow in limiting conditions [16,45,46,47], in both whole plant and in the derived calli a common response to stress is represented by the decrease in growth [48,49,50] and this is a typical drawback in the use of synthetic biotic elicitors and to a lesser extent for abiotic elicitors [51]. In this work, alteration in the fresh weight of ‘Spagnolo’ calli was monitored during 6 °C chilling stress treatment for 7 or 14 days or NaCl (50, 100, or 150 mM) salt stress treatment for 14 or 28 days, observing a strong reduction of fresh weight in a dose and time-dependent manner in salt stressed calli. Both salinity and low temperature induce osmotic alteration in plant cells, but salinity additionally creates ionic imbalance (i.e., excess sodium and associated potassium deficiency) [52], which could have exerted a stronger inhibition on the growth of cardoon calli than chilling. Opposite to NaCl-treated calli, the growth of chilled calli recovered after 14 days, possibly reflecting an osmotic adjustment through accumulation of osmolytes that usually keep cells turgid for growth [53]. In line with this observation, the value of lipid peroxidation revealed that chilling stress is successfully counterbalanced by the plant’s antioxidant machinery as suggested by the level of MDA that was even lower than the control. Though the discussion of these physiological aspects is beyond the scope of this work, we would like to point out that these eliciting strategies could be used for further study to understand mechanisms of cell adaptation to abiotic stresses.

Plant cells have evolved robust enzymatic and non-enzymatic mechanisms to buffer the outburst of ROS during stress. The antioxidant water-soluble phenolics are of main importance in non-enzymatic ROS scavenging mechanisms [54]. In cardoon, both salinity and low temperature triggered the same class of phenylpropanoids, and in particular hydroxycinnamic acids (CQA and *p*-coumaric acid derivatives), with salinity reaching higher levels than chilling stress in terms of metabolic enhancement of total phenols. This was observed both for calli and whole plants (this work and [16]). However, by comparing the results previously obtained for cardoon plants, it can be determined that flavonoids were induced more in whole cardoon plant than in calli [16]. We can speculate that the dark conditions, in which calli are grown, may be the reason for this divergence, in line with the hypothesis that flavonoids are produced during photoxidative stress [55]. Moreover, whereas plants have the possibility to delocalize the effect of stress [56,57,58], cells are directly exposed and different response mechanisms may be involved.

The research was further designed to directly identify the best growth conditions for obtaining an enriched extract with effective cosmeceutical potential. The skincare market has a growing interest in plant cell culture extracts since they represent a rich source of safe, sustainable and cruelty-free cosmetic ingredients. In cosmetics, the increasing interest toward “cosmeceutical” molecules, namely compounds having a cosmetic and pharmaceutical effect and lesser adverse reactions, increased the demand for bioactive phenylpropanoids and in particular for mono and dicaffeoylquinic acids [59,60,61,62]. Most of the chlorogenic acids for food supplements, pharmaceuticals, and cosmetic are recovered from coffee and teas, which are the most studied sources [63].

In two previous studies [15,16], we identified ‘Spagnolo’ an elective genotype accumulating flavonoids, benzoic acid derivatives but also the CGA. Interestingly, ‘Spagnolo’ calli accumulate abundant diesters of CQA (3,4-DiCQA and 1,5-DiCQA), while these compounds were undetectable in leaf extracts of three cardoon genotypes (including ‘Spagnolo’), even when plants were grown under salt stress [15]. Indeed, DiCQAs, which show stronger radical scavenging properties than mono-caffeoylquinic acids [64], are also less common than CQA, having a scattered distribution in a few plant families, including Solanaceae, Asteraceae, and Rubiaceae. As reported in *Coffea canephora*, DiCQAs might have a more transitory accumulation in leaves than CQA, because the former are more promptly degraded or exported to sustain other organs’ development [65,66]. Possibly, for this reason, the amount of detected DiCQAs may be variable according to leaf age and environmental and cultivation conditions. Several plant cell lines producing mono and diesters of CQA have been researched, including from artichoke as sister species [50,67,68], but to our knowledge, the potential of cardoon cell culture has been envisaged but not experimentally investigated. The successful elicitation of diCQAs by both salinity and chilling stresses we demonstrated in our study is an important step for developing novel platforms for stable production of these compounds that, together with CQA, contribute most to the radical scavenging ability of cardoon calli extracts, as shown by DPPH, ABTS, and FRAP assays. The use of different methods for measuring the antioxidant activity ensures robustness to our results, since an individual test can reflect only the chemical reactivity under the specific conditions applied for the assay. As previously reported [15], DPPH radical scavenging, ABTS decolorization, and FRAP assays are the most widely used methods due to their simplicity, stability, accuracy, and reproducibility. Our results, as expected, suggested a strict correlation between the increase in polyphenols and antioxidant power.

Given the recognized value of cardoon bioactive ingredients, the healthcare and cosmetic potential of hydroalcoholic cardoon calli extracts were evaluated by measuring their cytotoxicity, wound healing, and hydration power. To do so, firstly we selected which stress conditions showed the best eliciting power in terms of ability to boost phenylpropanoids and calli growth. Both NaCl and chilling stresses were able to induce the production of phenylpropanoids, albeit the eliciting effect of low temperature was less marked than salt stress. Nevertheless, salt stress imposition, although very efficiently rising phenylpropanoid production, severely impaired calli growth after 14 days at the highest salt concentration (150 mM) and at any concentration after prolonged stress (28 days). With the future perspective of using these elicitations for a scaled-up production of cardoon cells, we selected 100 mM NaCl as the best option in terms of growth parameters, and efficacy of metabolite production. The hydroalcoholic extracts of cardoon calli were safe as indicated by the cytotoxicity tests. The results of pro-collagen I production suggested that the higher the concentration of the bioactive compounds in the extract, the higher are the negative effects. In fact, extracts from unstressed calli showed a better potential than the elicited ones. The concentration of the major bioactive compounds of the extracts, namely CQA, 3,4 DiCQA and 1,5 DiCQA, may work positively only in a range of very low concentrations (below 0.002%). Similarly, the metabolic repertoire of elicited calli at 0.002% of the extract concentration works better than others as regards AQP3 induction [35,69,70]. Overall, cardoon cell extracts were able to act independently on different cell types that compose the main layers of the skin, by inducing collagen I and III synthesis in the fibroblasts and increasing the expression of genes related to hydration in the keratinocytes, through Aquaporin-3 (AQP3). Therefore, cardoon calli contains compounds capable of stimulating skin regeneration and hydration, thereby counteracting molecular pathways leading to skin damage and aging.

## 5. Conclusions

The results of this work sustain the possibility to valorize cardoon cells as bio-factories whose extracts can have a good biological potential for nutraceutical and cosmeceutical applications, building upon recent studies, also from our group, aimed at valorizing cardoon cell-based biorefinery in a different and sustainable way. For example, it was evidenced that cardoon cell cultures are suitable to be scaled up in bioreactors with the use of alternative organic carbon sources than sucrose (e.g., glucose, fructose, galactose, and starches). Moreover, it is possible to use biotechnological approaches to increase accessibility of the biomass to enzymatic degradation for an efficient “green extraction”. Finally, we evidenced that it is necessary to identify a correct working dilution for the cardoon cell extracts to benefit their ability to stimulate skin regeneration and hydration, thereby counteracting molecular pathways leading to skin damage and aging. Overall, these results contribute to provide the basis for the future development of an effective industrial production of higher yields of bioactive molecules from cardoon cell cultures, with reduced production costs and in an environmentally sustainable way.

## Figures and Tables

**Table 1:** Changes in polyphenol content measured by HPLC-MS analysis in cardoon callus cultures in response to 7 and 14 days of chilling stress at 6 °C. Each value represents the mean of three biological replicates ± SD. Statistical significance for each compound within each time point was determined by using one-way *ANOVA* with Tukey’s post hoc test. Different letters indicate significant difference (*p*-value < 0.05).

	25 °C	6 °C	25 °C	6 °C
Compounds	7 Days		14 Days	
	µg g^−1^ DW		µg g^−1^ DW	
3-CQA (CGA)	1.741 ± 0.141 b	34.299 ± 1.658 a	25.805 ± 1.630 b	33.113 ± 2.662 a
3-FQA	3.987 ± 0.155 b	5.621 ± 0.549 a	5.876 ± 0.628 a	5.988 ± 0.226 a
5-FQA	77.541 ± 1.417 b	112.655 ± 2.513 a	86.781 ± 3.452 b	93.098 ± 1.761 a
3,4-DiCQA	2543.67 ± 12.504 b	3512.788 ± 11.437 a	2865.411 ± 144.080 b	3276.89 ± 68.011 a
1,5-DiCQA	2346.451 ± 53.713 b	3562.412 ± 199.066 a	2755.342 ± 110.307 b	3145.453 ± 187.963 a
5-iFQA	17.766 ± 1.274 b	31.711 ± 0.740 a	21.432 ± 1.853 b	29.882 ± 2.208 a
*p*-coumaric acid	4.785 ± 0.186 b	16.341 ± 1.989 a	3.834 ± 0.270 b	8.999 ± 0.456 a
quercetin-glucoside	1.627 ± 0.278 b	5.36 ± 0.053 a	3.521 ± 0.282 a	1.117 ± 0.289 b
myricetin	N.d	N.d	N.d	N.d
kaempferolo-glucoside	1.226 ± 0.005 a	1.211 ± 0.156 a	1.226 ± 0.161 a	1.222 ± 0.157 a
naringin	1.431 ± 0.114 a	1.185 ± 0.097 b	0.989 ± 0.032 b	1.347 ± 0.130 a
vitexin	N.d	N.d	0.026 ± 0.008	0.014 ± 0.001
quercetin	0.277 ± 0.015 b	0.416 ± 0.006 a	0.137 ± 0.005 b	0.277 ± 0.039 a
diosmin	N.d	N.d	N.d	N.d
luteolin	1.746 ± 0.106 a	1.768 ± 0.137 a	1.741 ± 0.255 a	1.74 ± 0.133 a
kaempferol	3.666 ± 0.140 a	3.709 ± 0.379 a	3.641 ± 0.265 a	3.65 ± 0.257 a
apigenin	0.011 ± 0.002 b	0.024 ± 0.001 a	0.007 ± 0.001 a	0.007 ± 0.000 a
Total phenols	5005.980 ± 91.680 b	7289.527 ± 211.430 a	5775.769 ± 121.320 b	6602.797 ± 129.770 a

N.d., not detected; 3-CQA, 3-caffeoyl quinic acid (chlorogenic acid, CGA); 3-FQA, 3-feruloyl quinic acid; 5-FQA, 5-feruloyl quinic acid; 3,4-DiCQA, 3,4-dicaffeoyl quinic acid; 1,5-DiCQA, 1,5-dicaffeoylquinic acid (cynarin); 5-iFQA, 5-O-isoferuloylquinic acid.

**Table 2:** Changes in polyphenol content measured by HPLC-MS analysis in cardoon callus cultures in response to NaCl treatment (0, 50, 100, or 150 mM) for 14 or 28 days. Each value represents the mean of three biological replicates ± SD. Statistical significance for each compound among treatments within each time point was determined by using one-way ANOVA with Tukey’s post hoc test. Different letters indicate significant difference (*p*-value < 0.05).

	0 mM	50 mM NaCl	100 mM NaCl	150 mM NaCl	0 mM	50 mM NaCl	100 mM NaCl	150 mM NaCl
	14 Days				28 Days			
Compounds	µg g^−1^ DW				µg g^−1^ DW			
3-CQA (CGA)	28.944 ± 1.448 d	53.423 ± 1.531 c	81.650 ± 3.256 b	140.439 ± 2.647 a	8.315 ± 0.140 d	11.6015 ± 0.190 c	165.577 ± 3.163 b	263.123 ± 2.833 a
3-FQA	5.760 ± 0.274 b	5.987 ± 0.249 b	6.099 ± 0.109 ab	6.544 ± 0.250 a	5.154 ± 0.280 d	5.888 ± 0.00 c	6.032 ± 0.014 b	6.987 ± 0.190 a
5-FQA	85.445 ± 1.276 d	153.645 ± 4.728 b	134.987 ± 2.325 c	165.981 ± 3.746 a	81.876 ± 0.629 c	142.660 ± 0.00 b	145.898 ± 3.643 b	187.977 ± 0.786 a
3,4-DiCQA	2653.866 ± 7.959 d	2867.987 ± 3.425 c	4087.871 ± 142.984 b	4545.566 ± 112.699 a	2438.866 ± 18.321 c	5127.761 ± 16.971 b	5523.761 ± 412.001 b	6287.980 ± 29.602 a
1,5-DiCQA	2433.451 ± 1.724 d	2687.342 ± 46.400 c	3942.544 ± 93.496 b	4312.232 ± 12.736 a	2232.341 ± 141.293 c	5012.544 ± 165.614 b	5171.671 ± 225.069 b	6023.561 ± 120.024 a
5-iFQA	19.566 ± 0.789 c	21.688 ± 2.056 bc	24.642 ± 1.840 b	27.828 ± 0.697 a	14.761 ± 0.214 d	19.877 ± 0.502 c	24.882 ± 2.073 b	31.761 ± 0.949 a
p-coumaric acid	4.556 ± 0.332 c	12.559 ± 3.121 b	9.461 ± 1.372 b	27.552 ± 1.585 a	7.747 ± 0.135 d	9.926 ± 0.561 c	12.789 ± 0.767 b	16.500 ± 1.667 a
quercetin-glucoside	2.911 ± 0.537 d	3.792 ± 0.054 c	8.174 ± 0.052 b	9.500 ± 0.253 a	3.439 ± 0.272 c	6.641 ± 0.140 b	8.412 ± 0.129 a	7.199 ± 1.055 a
myricetin	N.d	N.d	N.d	N.d	N.d	N.d	N.d	N.d
kaempferolo-glucoside	1.216 ± 0.306 a	1.308 ± 0.161 a	1.219 ± 0.017 a	1.205 ± 0.023 a	1.254 ± 0.032 a	1.222 ± 0.137 a	1.213 ± 0.143 a	1.206 ± 0.135 a
naringin	1.655 ± 0.093 a	1.310 ± 0.009 b	1.176 ± 0.082 c	0.806 ± 0.148 d	0.868 ± 0.075 b	0.767 ± 0.123 b	1090 ± 0.215 ab	1151 ± 0.048 a
vitexin	0.024 ± 0.002 b	0.030 ± 0.004 a	0.013 ± 0.001 c	0.004 ± 0.002 d	0.022 ± 0.028 a	0.003 ± 0.000 c	0.010 ± 0.001 b	0.005 ± 0.001 c
quercetin	0.225 ± 0.135 ab	0.272 ± 0.010 a	0.075 ± 0.011 c	0.149 ± 0.015 b	0.240 ± 0.086 a	0.216 ± 0.049 a	0.151 ± 0.001 c	0.182 ± 0.001 b
diosmin	N.d	N.d	N.d	N.d	N.d	N.d	N.d	N.d
luteolin	1.737 ± 0.197	1.739 ± 0.038	N.d	N.d	1.736 ± 0.176 a	1.735 ± 0.002 a	N.d	N.d
kaempferol	3.626 ± 0.050 a	3.613 ± 0.281 a	3.610 ± 0.144 a	3.604 ± 0.011 a	3.619 ± 0.032 a	3.618 ± 0.133 a	3.616 ± 0.432 a	3608 ± 0.037 a
apigenin	0.004 ± 0.00 a	0.004 ± 0.00 a	N.d	0.001 ± 0.00 b	0.003 ± 0.00 a	0.002 ± 0.00 a	0.002 ± 0.00 a	0.001 ± 0.00 a
Total phenols	5242.985 ± 120.210 d	5814.700 ± 162.332 c	8301.521 ± 234.433 b	9241.412 ± 231.220 a	4800.243 ± 132.440 d	10,448.876 ± 123.331 c	11,065.104 ± 354.540 b	12,831.239 ± 121.433 a

N.d., not detected; 3-CQA, 3-caffeoyl quinic acid (chlorogenic acid, CGA); 3-FQA, 3-feruloyl quinic acid; 5-FQA, 5-feruloyl quinic acid; 3,4-DiCQA, 3,4-dicaffeoyl quinic acid; 1,5-DiCQA, 1,5-dicaffeoylquinic acid (cynarin); 5-iFQA, 5-O-isoferuloylquinic acid.

**Table 3:** Lipid peroxidation of cardoon calli in response to 7 and 14 days of chilling at 6 °C or 14- and 28 days of 0, 50, 100, or 150 mM NaCl stress, as measured by the MDA assay. Each MDA value (μmol g^−1^ FW) represents the mean of three biological replicates ± SD. Statistical significance between treatments within each time point was determined by one-way ANOVA with Tukey’s post hoc test. Different letters indicate significant differences (*p*-value < 0.05).

Treatments		MDA Value μmol g^−1^ FW	
		7 days	14 days
Control	25 °C	4.404 ± 0.357 a	4.679 ± 1.018 a
Chilling Stress	6 °C	3.544 ± 0.215 b	3.785 ± 0.413 a
		14 days	28 days
Control	0 mM	3.578 ± 0.705 b	3.475 ± 0.677 b
Salt stress (NaCl)	50 mM	3.939 ± 0.775 ab	5.012 ± 0.559 a
	100 mM	4.955 ± 0.477 a	5.058 ± 1.424 ab
	150 mM	5.5651 ± 0.921 a	6.664 ± 1332 a

## Data Availability

Data is contained within the article or Supplementary Material.

## Supplementary Materials

The following supporting information can be downloaded at: https://www.mdpi.com/article/10.3390/antiox11061041/s1, Figure S1: (A) Growth of cardoon calli upon 28 days subculturing on solid GB5 medium (B) Liquid growth of cardoon calli after subculturing in a fresh liquid medium (left side) and upon 28 days of growth in liquid GB5 medium (right side).
