# Supplementary material for: Abiotic Stresses Elicitation Potentiates the Productiveness of Cardoon Calli as Bio-Factories for Specialized Metabolites Production
Source: Antioxidants (Basel). 2022 May 24;11(6):1041. doi: 10.3390/antiox11061041 (PMC9219710; doi:10.3390/antiox11061041)
Supplement: Supplementary file 1 [file antioxidants-11-01041-s001.zip › antioxidants-1730910-supplementary.pdf]

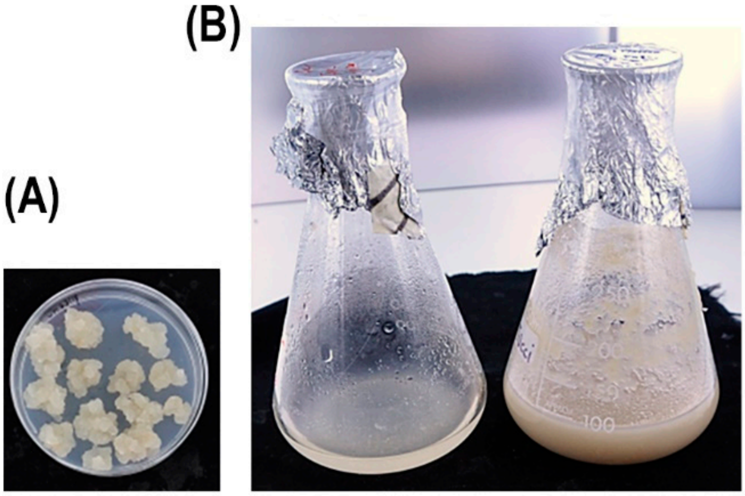

Figure S1. A-B. (A) Growth of cardoon calli upon 28 days subculturing on solid GB5 medium (B) Liquid growth of cardoon calli after subculturing in a fresh liquid medium (left side) and upon 28 days of growth in liquid GB5 medium (right side).
